# Prediction of unexpected B_*n*_P_*n*_ structures: promising materials for non-linear optical devices and photocatalytic activities†

**DOI:** 10.1039/d0na01040e

**Published:** 2021-03-26

**Authors:** Zabiollah Mahdavifar

**Affiliations:** Department of Chemistry, Faculty of Science, Shahid Chamran University of Ahvaz Ahvaz Iran z_mahdavifar@scu.ac.ir +98-611-3331042

## Abstract

In the present work, a modern method of crystal structure prediction, namely USPEX conjugated with density functional theory (DFT) calculations, was used to predict the new stable structures of B_*n*_P_*n*_ (*n* = 12, 24) clusters. Since B_12_N_12_ and B_24_N_24_ fullerenes have been synthesized experimentally, it motivated us to explore the structural prediction of B_12_P_12_ and B_24_P_24_ clusters. All new structures were predicted to be energetically favorable with negative binding energy in the range from −4.7 to −4.8 eV per atom, suggesting good experimental feasibility for the synthesis of these structures. Our search for the most stable structure of B_*n*_P_*n*_ clusters led us to classify the predicted structures into two completely distinct structures such as α-B_*n*_P_*n*_ and β-B_*n*_P_*n*_ phases. In α-B_*n*_P_*n*_, each phosphorus atom is doped into a boron atom, whereas B atoms form a B_*n*_ unit. On the other hand, each boron atom in the β-phase was bonded to a phosphorus atom to make a fullerene-like cage structure. Besides, theoretical simulations determined that α-B_*n*_P_*n*_ structures, especially α-B_24_P_24_, show superior oxidation resistance and also, both α-B_*n*_P_*n*_ and β-B_*n*_P_*n*_ exhibit better thermal stability; the upper limit temperature that structures can tolerance is 900 K. The electronic properties of new compounds illustrate a higher degree of absorption in the UV and visible-region with the absorption coefficient larger than 10^5^ cm^−1^, which suggests a wide range of opportunities for advanced optoelectronic applications. The β-B_*n*_P_*n*_ phase has suitable band alignments in the visible-light excitation region, which will produce enhanced photocatalytic activities. On the other hand, α-B_*n*_P_*n*_ structures with modest band gap exhibit large second hyperpolarizability, which are anticipated to have excellent potential as second-order non-linear optical (NLO) materials.

## Abstract 2

In the present work, a modern method of crystal structure prediction, namely USPEX conjugated with density functional theory (DFT) calculations, was used to predict the new stable structures of B_*n*_P_*n*_ (*n* = 12, 24) clusters.

## Graphical abstract

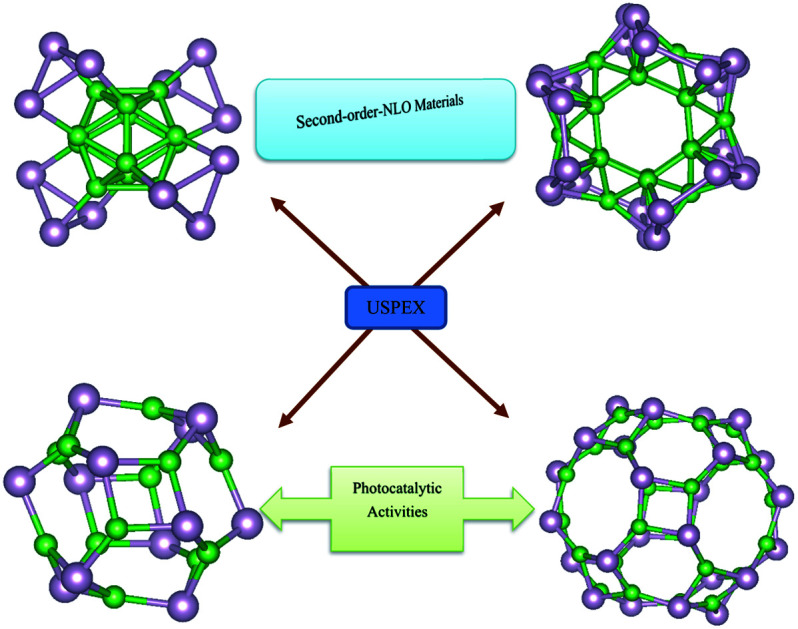

## 1. Introduction

In the past decade, many attempts have been made to discover non-carbon fullerene-like structures based on their unique properties.^1–9^ The structural and electronic properties of clusters form an intermediate state between the individual atoms and crystals. Clusters have a remarkable impression in understanding the chemical bonding and the rational design of molecules with tailored physicochemical properties. In recent years, scientists have focused on studying boron-based materials because a significant number of unfamiliar structures of boron and its compounds have been explored.^10–16^ The electron configuration of boron is 2s^2^ 2p^1^; therefore, boron with one electron less than carbon has different characteristics compared to carbon, which can form fascinating and strong bonds with nearly all periodic table elements.^16–18^ On the other hand, phosphorus clusters with large pore sizes have received much attention in the past decade.^18^ Phosphorus is the diagonal neighbor of carbon in the periodic table; therefore, the chemical bonding and structural chemistry of phosphorus are also interesting. Elemental phosphorus exists in different forms such as white, yellow, and black phosphorus but phosphorus is never found as a free element on earth because of its high reactivity.^19^

The group III–V compounds such as boron phosphide (BP) have been most generally investigated in their cubic crystal phase (c-BP).^20^ Also, BP compounds can exist in planar (two-dimensional), tubular (one-dimensional), or spherical shapes (zero-dimensional), quite the same as graphene, carbon nanotubes, and fullerenes, respectively. All structures of BP compounds are prominent as large band-gap semiconductor materials having many advanced applications including microelectronics and optoelectronics,^21,22^ high thermal conductivity^23^ hardness, chemical stability,^23–25^ and catalysis.^20,26,27^ Both boron and phosphorus elements can form 2D materials; it is interesting to determine whether the binary compound of boron and phosphorus can form stable clusters, which may display unusual structural and electronic properties better than both boron and phosphorus clusters.^28^ Hence, it is desirable to consider the structures of boron phosphide clusters with different sizes.

Numerous computational and experimental studies have considered the structural and electronic properties of group III–V compounds.^18,19,29–32^ In recent years, non-carbon (inorganic) (XY)_*n*_ fullerenes of group III–V are of great scientific interest based on their promising candidates as optoelectronic devices^33,34^ and light-emitting diodes (LEDs).^35^ X_12_Y_12_, X_16_Y_16_, X_24_Y_24_, and X_28_Y_28_ clusters, where X = group III atom and Y = group V atom, are predominant inorganic (XY)_*n*_ fullerenes.^8^ Computational investigations on (XY)_*n*_ fullerenes have demonstrated that (XY)_12_ has the highest thermodynamic stability, which is known as a magic cluster.^36–38^ In this regard, Strout *et al.* ascertained that the X_12_Y_12_ nano-cages are the most stable structures between all fullerene-like (XY)_*n*_ (X = B, Al, Ga,… and Y = N, P, As,…) structures.^36^ B_12_N_12_ and B_24_N_24_ fullerene-like clusters were synthesized by Oku *et al.*^39–41^ and detected using time-of-flight mass spectrometry. The B_12_N_12_ cluster is shown as the most stable structure among B_12_N_12_, B_16_N_16_, and B_28_N_28_.^42^ Other (XY)_12_ clusters such as Al_12_N_12_, Al_12_P_12_, B_12_N_12_, and B_12_P_12_ are of much interest owing to their unique chemical and physical properties. In this regard, many theoretical studies had been focused on ascertaining the relative stabilities of different sizes of these nano-cages.^18,19,30–32,38,43^

Boron phosphide (BP)_*n*_ clusters are an attractive member of the X_*n*_Y_*n*_ nano-cage family with unique physical and electrochemical properties. Since B_12_N_12_ and B_24_N_24_ fullerenes have been synthesized experimentally,^39–41^ it has motivated researchers to investigate the structural and electronic properties of B_12_P_12_ and B_24_P_24_ fullerene-like clusters. In all the previous studies, X_12_Y_12_ and X_24_Y_24_ clusters have been considered as fullerene-like structures, which consist of four-, six-, and eight-membered rings, and have large HOMO–LUMO gaps, almost zero dipole moment, and nearly zero hyperpolarizability.^31^ It should be mentioned that in all the previous studies, (XY)_*n*_ clusters (X = B, Al, Ga, and Y = P, As) were made directly by replacing B and N atoms of the already known structures of the (BN)_*n*_ cages with other atoms, then the bonding length and angle are adjusted by further calculations. Based on this strategy, the main questions are that whether this method is correct and whether this method give the lowest energy structure. We believe that structural prediction can answer these questions. Structural prediction based on first-principles calculation has served as a very useful tool in materials research. Exploring 0, 1, and 2-dimensional (0, 1, 2-D) materials from molecular design and global search have been a hot-topic. Based on our knowledge, no study has been conducted to predict the structure of boron phosphide (BP)_*n*_ clusters. Also, all previous studies on these materials have been based on the structure of boron nitride (BN)_*n*_ clusters. The structure of B_*n*_P_*n*_ in all previous investigations by other researchers has been reported to be cage-shaped, which is exactly similar to the (BN)_*n*_ clusters, in which the P atoms are replaced by N atoms. It motivated us to use an evolutionary algorithm to predict the structures of boron phosphide clusters. For the first time, we used the evolutionary algorithm as implemented in the USPEX code to search the ground state structure of the B_*n*_P_*n*_ clusters, including B_12_P_12_ and B_24_P_24_ clusters. It should be noted that the B_12_N_12_ and B_24_N_24_ fullerenes have been synthesized experimentally; hence, the B_12_P_12_ and B_24_P_24_ structures were chosen.

## 2. Computational details

We used the evolutionary algorithm as executed in the USPEX code to search for the potential energy surface of the B_12_P_12_ and B_24_P_24_ clusters. USPEX code by evolutionary algorithm solves the central problem of crystal structure prediction of theoretical crystal chemistry.^44–47^ An adequate number of initial structures, more than 1800 configurations in 50 generations by the USPEX method, was used to ensure that the global minimum is attained. The geometrical relaxation, stability assessment, and optoelectronic properties of the predicted B_*n*_P_*n*_ clusters were carried out by the Vienna *ab initio* simulation package (VASP).^48,49^ The generalized gradient approximation (GGA) scheme, developed by Perdew–Burke–Ernzerhof (PBE) and projector-augmented wave (PAW)^50^ potentials were used. The dispersion-corrected DFT-D3 method with Beack-Jansoon damping was used in all the structure optimizations to account for the weak van der Waals interactions.^51,52^ The low-lying energy isomers, whose relative energies are in the range of 0–2.5 eV with respect to the most stable structure, were carefully re-optimized to distinguish the correct global minimum structure of each B_*n*_P_*n*_ cluster. The screen hybrid HSE06 method^53^ with the mixing parameter alpha value of 0.25 was carried out to consider the electronic properties and optical absorption of the predicted clusters. An energy cutoff of 400 eV was used for the plane-wave expansion. All geometries were relaxed until the convergence criteria of energy (10^−6^ eV) and force on each atom (0.02 eV Å^−1^) were satisfied. It should be pointed out that the structures were located in the middle of a box with a vacuum spacing of ∼10 Å in all the directions to neglect the interactions between the adjacent cells. To further examine the thermal stability proposed for the B_*n*_P_*n*_ clusters, *ab initio* molecular dynamics (AIMD) simulations using the canonical (NVT) ensemble were performed. The simulations are accomplished using a Nosé–Hoover thermostat at finite 300, 600, and 900 K temperatures for 5000 fs with a time step of 1.0 fs.

## 3. Results and discussion

### 3.1. Geometry and stability

**Fig. 1:**
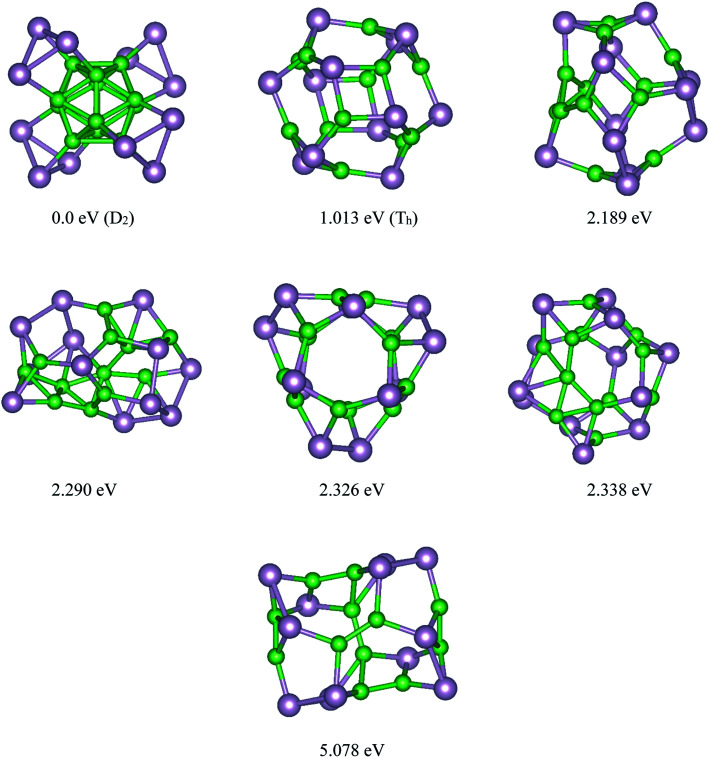
Lowest-energy structures and low-lying energy isomers of the P_12_B_12_ clusters. The green and gray balls represent the B and P atoms, respectively.

**Fig. 2:**
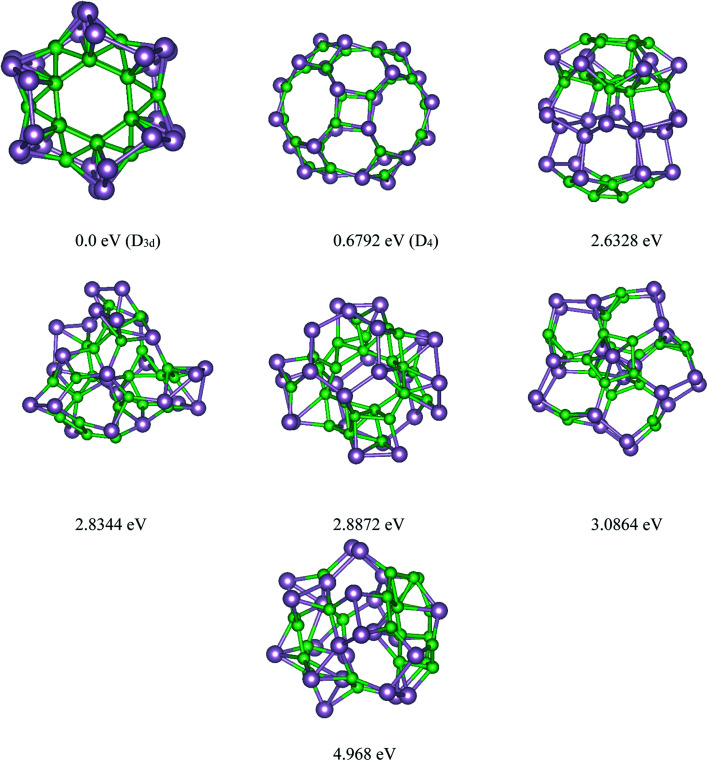
Lowest-energy structures and low-lying energy isomers of the P_24_B_24_ clusters. The green and gray balls represent the B and P atoms, respectively.

Based on the USPEX method conjugated with spin-polarized first-principles calculations, a comprehensive study to explore their ground state structures of B_*n*_P_*n*_ (*n* = 12, 24) and low-lying isomers of these clusters was performed employing the evolutionary algorithm. The USPEX method has been successful used before to explore a wide range of materials.^54–56^ Among the structure search by evolutionary algorithm, more than 1800 different structures for each cluster were predicted. We mainly focused on the low-lying isomers in the energy range of 0–2.5 eV with respect to the most stable structure. We re-optimized these structures to get the precise total energy and the atomic structures. The lowest-energy structures and low-lying energy isomers of the B_*n*_P_*n*_ (*n* = 12, 24) clusters are displayed in Fig. 1 and 2.

Our search for the most stable structure of B_*n*_P_*n*_ clusters led us to classify the predicted structures into two completely distinct structures. Interestingly, it can be classified into two α- and β-phases. In the α-phases, each phosphorus atom is doped into a boron atom while the B atoms form a B_*n*_ unit. This type of predicted structure named α-B_*n*_P_*n*_ structure, is reported for the first time. On the other hand, in the β-phase, each boron atom is bonded to each phosphorus atom to make a cage cluster, which is named the β-B_*n*_P_*n*_ structure. The final relaxed geometry of the α-B_*n*_P_*n*_ and β-B_*n*_P_*n*_ systems with their symmetries, their low-lying energy isomers, and relative stabilities are displayed in Fig. 1 and 2. To assist comprehension, two different views of the ground-state structures, as well as the first low-lying energy isomers of α-B_*n*_P_*n*_ and β-B_*n*_P_*n*_ are shown in Fig. S1 and S2,† respectively.

Based on our global structure search, the most stable structure of the B_12_P_12_ cluster with *D*_2_ symmetry is alpha rhombohedral-shaped, named as α-B_12_P_12_. In this structure, the arrangement of 12 boron atoms in the alpha-tetragonal boron structure made a B_12_ building block, while each phosphorus atom bonded to each boron atom of the alpha tetragonal B_12_ unit. To date, this is the first time that this type of structure has been reported for the B_12_P_12_ structure. In this beautifully predicted structure, each P atom is covalently bonded to each boron atom of the B_12_ unit with an average B–P bond length of ∼1.98 Å. In other words, the surface of the B_12_ unit is covered with phosphorus atoms. The average B–B bond lengths in the B_12_ unit of α-B_12_P_12_ (∼1.77 Å) is nearly the same as that of the average B–B bond in the B_12_ icosahedron (1.75 Å).^57^ As seen in Fig. 1, each phosphorus atom is covalently bonded to two neighboring phosphorus atoms to make a three-membered ring, in which the phosphorus atoms are lying in a plane. It can be imagined that the triangular rings of phosphorus have grown on the B_12_ unit, while each of these P atoms are bonded to each of the B atoms. In this situation, the hybridization of each P atom is sp^3^ The calculated average P–P bond length and the P–P–P bond angles of α-B_12_P_12_ are about 2.36 Å and 60°, respectively, which is comparable to the P–P bond length (2.23 Å) in phosphorene.^58^ The experimental evidence for our predicted α-B_12_P_12_ structure is the alpha tetragonal allotrope of boron determined in 1951 by Hoard *et al.*^59^ The same as α-B_12_P_12_ structure, this structure is made up of just one building block of the alpha-tetragonal B_12_ boron structure. Another evidence for the new predicted structure is B_13_P_2_.^60^ The crystal structure of B_13_P_2_ shows that this is an alpha rhombohedral boron structure with two P atoms in the unit cell. Interestingly, B_13_P_2_ was made up of just one B_13_ building block, in which P atoms connected each building block.^60^

The first low-lying energy structure of B_12_P_12_ is fullerene-like with a large cavity. This type of structure was named as the β-B_12_P_12_ structure. The structure of β-B_12_P_12_ is a polyhedral boron-phosphide structure composed of four tetragons and hexagons. As seen in Fig. 1, the stability of the low-lying energy β-B_12_P_12_ structure is less than the α-B_12_P_12_ configuration with a large amount of energy of about 1.013 eV. Based on our calculations, it has an average diameter of 0.586 nm, while the C_60_ fullerene has an average diameter of ∼0.7 nm.^61,62^ Due to the large cavity of β-B_12_P_12_, the same as that of C_60_ fullerene, it can be suitable for various applications such as gas storage, encapsulation of atoms, or molecules inside its cage, and endohedral metallofullerenes. As we know, the endohedral metallofullerenes have attracted much attention due to their unique potential applications such as superconductors and non-linear optical (NLO) devices. The relaxed geometry of the β-B_12_P_12_ cluster indicated that only B–P bonds with an average bond length of ∼1.911 Å were found in this nano-cage. It should be noted that two types of B–P bonds were found in this structure; the first is the B–P bond in the hexagonal ring, which is named I (B–P), and the second is the B–P bond in the tetragonal rings, which is named II (B–P). These results are collected in Table 1 and are shown in Fig. S1.†

**Table 1:** The PBE-D3 structural parameters of the relaxed geometries of the most stable structures and the first low-lying energy isomers of the (PB)_*n*_ clusters (atom numbering is in accordance with see ESI Fig. S1 and S2)

Type	B–P (Å)			P–P (Å)	B–B (Å)	
	I(B–P)_h_	II(B–P)_T_	III(B–P)_O_		I(B–B)_h_	II(B–B)_Tr_
α-B_12_P_12_	1.98	—	—	2.36	1.77	—
β-B_12_P_12_	1.900	1.922	—	—	—	—
α-B_24_P_24_	1.972	2.012	—	2.231	1.746	1.788
β-B_24_P_24_	1.918	1.905	1.871	—	—	—

The final relaxed geometry of the ground-state structure and the low-lying isomers of the B_24_P_24_ cluster, together with their symmetries and relative stabilities, are shown in Fig. 2 and S2.† By looking at the predicted structures of B_24_P_24_, it was found that the ground state structure of B_24_P_24_ is α-B_24_P_24_ with a high symmetry of *D*_3d_, in which all the boron atoms are arranged as B_24_ unit, while the phosphorus atoms cover the B_24_ surface. It should be pointed out that the B_24_ motif in α-B_24_P_24_ is composed of two hexagons on the bottom and the top, six hexagons surrounding the waist, and 12 trigons alternatively connected to each hexagon. To date, this has been the first time that this type of structure has been reported for the P_24_B_24_ cluster. In this unique predicted structure, each P atom is bonded to one or more boron atoms of the B_24_ motif as the surface of the B_24_ unit is covered with phosphorus atoms. The average B–P bond length is obtained as ∼1.99 Å, which is comparable with the B–P bonds in phosphinoboranes and related compounds.^63^ In detail, two types of B–P bonds such as types I and II can be found in the α-B_24_P_24_ structure (Fig. S2†), and the corresponding values of these bond lengths are collected in Table 1. On the other hand, the calculated values of the P–P and B–B bond lengths are summarized in Table 1. The average P–P bond length is obtained as 2.231 Å while for the B–B bonds, we found two different types of B–B in *τηε* α-B_24_P_24_ structure. I (B–B)_h_ represents the bonded B atoms to each other in hexagons (1.746 Å) and II (B–B)_T_ shows the bond length between the boron atoms in trigonal geometry with a bond length of about 1.788 Å. These results are comparable with the average-B–B bond in the B_12_ icosahedra (1.75 Å).^57^ For simplicity of visualization, two different views of the B_24_ unit without P atoms are shown in Fig. S3.† This result is contradictory to that reported for pristine B_24_ clusters, where the quasi-planar and rather irregular polyhedrons are prevalent.^64^ It should be reminded that the ionic quasi-planar B_24_ structure has been experimentally synthesized.^64^ The evidence for our predicted α-B_24_P_24_ cluster is the encapsulation of a transition metal in the fullerene-like boron cages.^65–67^ Yanming Ma and coworkers^65^ predicted transition metal-doped B_24_ clusters using first-principles swarm-intelligence-based structure searches. They found that the low-lying energy structures were generally a cage-like structure. It can be concluded that doping is a significant factor in determining the geometry of boron-based materials.

Fig. 2 represents the selected low-lying energy B_24_P_24_ structures with their symmetry and energy relative to the ground-state *D*_3d_ α-B_24_P_24_ structure. The first low-lying energy structure belongs to the β-B_24_P_24_ fullerene-like structure. As seen from this figure, the β-B_24_P_24_ structure with *D*_4_ symmetry is less stable than the ground-state α-B_24_P_24_ structure by a large amount of energy (∼0.68 eV). It should be reminded that the fullerene-like structure is proposed as the ground-state structure of the X_24_Y_24_ clusters. The investigated structures of X_24_Y_24_ in all the previous studies^19,30–34,38,43^ are based on the strategy in which B and N atoms are directly replaced with X and Y atoms in the already known structures of the (BN)_*n*_ nano-cages, and then the bonding length and angle are adjusted for further calculation. In the case of B_*n*_P_*n*_, our global search indicated that the B_*n*_P_*n*_ fullerene-like structure is a low-lying isomer. The β-B_24_P_24_ fullerene-like structure with a 0.859 nm cavity, which is larger than the C_60_ cavity (0.7 nm), includes 12 tetragonal, eight hexagonal, and six octagonal BP rings. Table 1 summarizes different boron–phosphorus (B–P) bond lengths of the most stable structures and the first low-lying energy structures of B_24_P_24_. As can be seen in Fig. 2 and Table 1, two kinds of B–P bonds are recognized for the β-B_24_P_24_ structures with B–P bonds between tetragonal/hexagonal rings, named type I (B–P), with an average bond length of about 1.918 Å, B–P bonds between tetragonal/octagonal rings, named as type II (B–P) (∼1.905 Å), and the B–P bonds between hexagonal/octagonal ring, named type III, with bond length of about 1.871 Å.

The energy difference between the most stable structure and other competing structural isomers of each compound are essential to figure out the possibility of their synthesis under different experimental conditions. The energy difference between the lowest energy structure of α-B_12_P_12_/α-B_24_P_24_ and the first low-lying energy β-B_12_P_12_/β-B_24_P_24_ structures is 0.059/0.061 eV per atom, respectively, implying that the α-B_*n*_P_*n*_ structures are more stable than the β-B_*n*_P_*n*_ structures (≥5.7 kJ mol^−1^). Based on our predictions, β-B_12_P_12_ and β-B_24_P_24_ clusters may not coexist with the α-B_*n*_P_*n*_ structures at room temperature when compared with the thermal energy at 300 K (0.026 eV). Hence, it can be concluded that the B_*n*_P_*n*_ fullerene-like structures can exist at higher temperatures (>300 K). In the case of other low-lying energy isomers of B_12_P_12_ and B_24_P_24_ structures, the second and third metastable structures are less stable than the global minimum α-B_12_P_24_ and α-B_24_P_24_ structures by a large amount of energy (>2.2 eV).

Now, we return to investigate the thermodynamic stability of the α-B_*n*_P_*n*_ and β-B_*n*_P_*n*_ clusters, which need to certify their stability and facility experimental synthesis. To estimate the thermodynamic stability of the predicted clusters, the binding energy per atom (*E*_b_, eV per atom) is calculated. The relative binding energy (*E*_b_) is an effective parameter to show the thermodynamic stability of the clusters and it is defined as below.

1 *E*_bin_ = [*E*_(BP)n_ − *n*/2(*E*_B_ + *E*_P_)]/*n*

where *E*_(BP)_*n*__ and *E*_B_/*E*_P_ illustrate the total energies of the B_*n*_P_*n*_ clusters and a single boron/phosphorus atom, respectively.

As seen in Table 2, the calculated binding energies of the α-B_*n*_P_*n*_ and β-B_*n*_P_*n*_ structures are in the range from −4.723 to −4.803 eV per atom, which is much better or comparable to that of other 2D materials such as phosphorene,^68^ carbon phosphide,^69^ Al_2_C sheet,^70^ and silicene,^71^ having 3.48, 5.32, 3.94, and 3.55 eV per atom, respectively, indicating the very good stability of these materials. Furthermore, the binding energy of the predicted clusters was compared with that of different 2D-BP allotropes. The calculated binding energies for the α-B_*n*_P_*n*_ and β-B_*n*_P_*n*_ structures is comparable with the binding energy of 2D *α*_0_-BP (4.91 eV per atom), *α*_1_-BP (4.82 eV per atom), *β*_0_-BP (4.72 eV per atom), *β*_1_–BP (4.54 eV per atom), and *γ*-BP (4.44 eV per atom).^72^ Moreover, the binding energies for these new configurations are even close to the already synthesized B_40_,^73^ M@B_*n*_,^66^ and B_24_ clusters.^64^ Hence, it can be concluded that the α-B_*n*_P_*n*_ and β-B_*n*_P_*n*_ structures have excellent stability and possible experimental synthesis. As seen in Table 2, the α-B_*n*_P_*n*_ structures are more stable than the corresponding β-B_*n*_P_*n*_ structure and the maximum binding energy of −4.803 eV per atom belongs to the α-B_24_P_24_ structure.

**Table 2:** Symmetries, calculated binding energy per atom (*E*_bin_, eV per atom), HOMO–LUMO energy gaps (*Δ*_g_, eV), and VBM and CBM (eV) of the (BP)_*n*_ clusters. The structural and energetic properties are calculated using the PBE-D3 method, while the HSE06 functional was used to calculate the electronic properties (*Δ*_g_), VBM, CBM, and summation Bader partial charge on all B and P atoms (esu) of the HSE06 functional based on the PBE-D3 relaxed structures

Type	α-B_12_P_12_	β-B_12_P_12_	α-B_24_P_24_	β-B_24_P_24_
HOMO (eV)	−5.563	−6.186	−4.315	−5.572
LUMO (eV)	−4.895	−2.977	−3.691	−2.592
VBM (eV)	−6.120	−6.787	−5.313	−6.311
CBM (eV)	−5.532	−3.579	−4.689	−3.331
*E*_g_ (eV)	0.668	3.208	0.624	2.98
*E*_b_ (eV per atom)	−4.782	−4.723	−4.803	−4.742

**Total partial charge (esu) on all B and P atoms**				
B	0.577	3.555	2.313	8.242
P	−0.577	−3.555	−2.313	−8.242

### 3.2. Dynamic and chemical stability

**Fig. 3:**
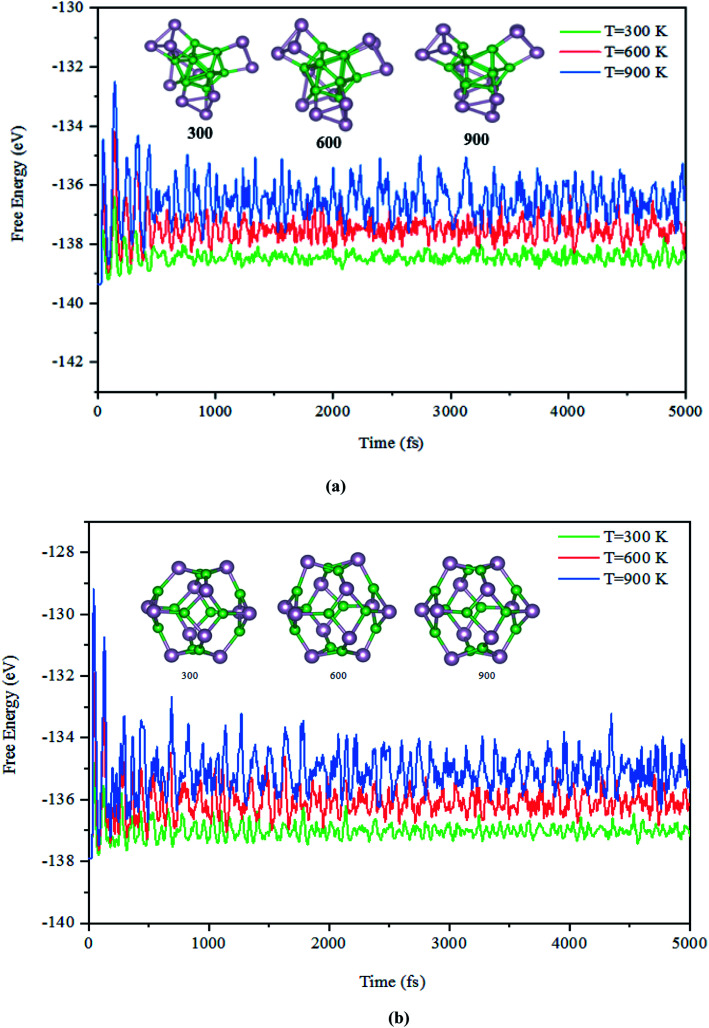
The snapshots of the most stable structures for a time of 5 ps of (a) α-B_12_P_12_ and (b) β-B_12_P_12_. The temperature of the AIMD was set to 300, 600, and 900 K.

**Fig. 4:**
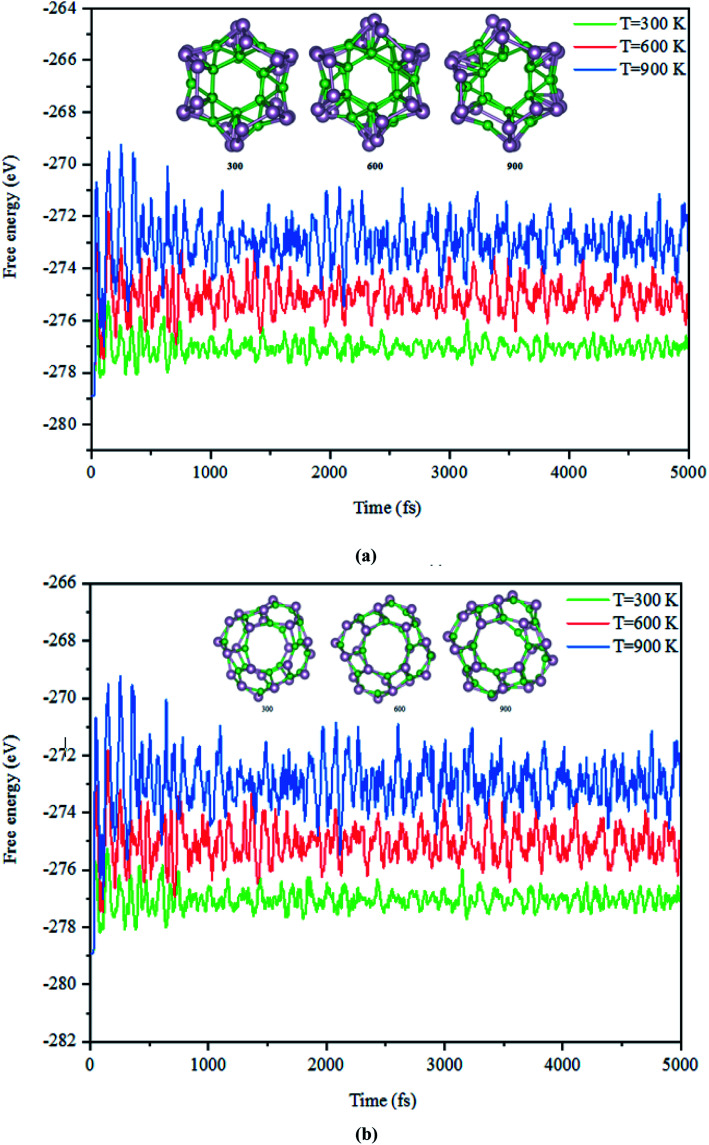
The snapshots of the most stable structures for a time of 5 ps of (a) α-B_24_P_24_ and (b) β-B_24_P_24_. The temperature of the AIMD was set to 300, 600, and 900 K.

Next, the dynamic stability of the predicted clusters was also investigated. The vibrational frequencies at the gamma points for the most stable structures as well as the first low-lying isomers were calculated. Based on these calculations, no negative vibration frequency could be found, confirming that the most stable and the first low-lying structures are dynamically stable. Furthermore, the thermal stability of the most stable structure and the first low-lying energy isomers of the studied B_*n*_P_*n*_ clusters was performed using *ab initio* molecular dynamic simulations (AIMD) at three different (300, 600, and 900 K) temperatures by the NVT-ensemble with the Nosé–Hoover thermostat. As represented in Fig. 3 and 4, the predicted structures equilibrated at 300 K from the AIMD simulations. Furthermore, there is no broken bond and the initial clusters are well maintained during the AIMD simulation for 5 ps with a time-step of 1 fs. As shown in these figures, the total potential energy only fluctuates around a certain constant magnitude, which indicates the most stable structures of the B_*n*_P_*n*_ clusters and the first low-lying isomers are thermodynamically stable and could hold their structural integrity at room temperature. It can be seen from Fig. 3 and 4 that after heating both α-B_*n*_P_*n*_ and β-B_*n*_P_*n*_ structures, the basic structure of the clusters is also well maintained at 600 and 900 K. Furthermore, the thermal stability of the α-B_24_P_24_ cluster at a higher temperature such as 1200 K was also examined (Fig. S4†). Based on the AIMD simulations, when the temperature reaches as high as 900 K, the bond elongation of the 6-membered ring occurs. It can be anticipated that as the temperature increases, bond stretching is also increased. Hence, the upper limit of temperature that the structures can tolerate is 900 K and the bond stretching occurs between 900 and 1200 K.

Chemical stability in air and other environments is an important issue that may limit the future applications of materials. Furthermore, a major problem with atoms located on the surface of the materials is their reaction with oxygen, nitrogen, and water in air; hence, the surface chemistry of these materials is essential in determining the intrinsic properties.^74,75^ Some phosphorus-based materials such as 2D-phosphorene and black phosphorus are known to oxidize and break down in ambient conditions because of the lone atomic pairs of the P atoms at the surface.^76–80^ Generally, P atoms favor the formation of the trigonal pyramidal sp^3^ bonded configuration.^81^ It is found that in the α-B_12_P_12_ cluster, each P atom with sp^3^ hybridization bonding with two P and one B neighbor atoms has one lone pair of electrons; hence, it motivates us to investigate the effects of oxygen, nitrogen, and water–gas molecules on the stability of the α-B_*n*_P_*n*_ structures.

**Fig. 5:**
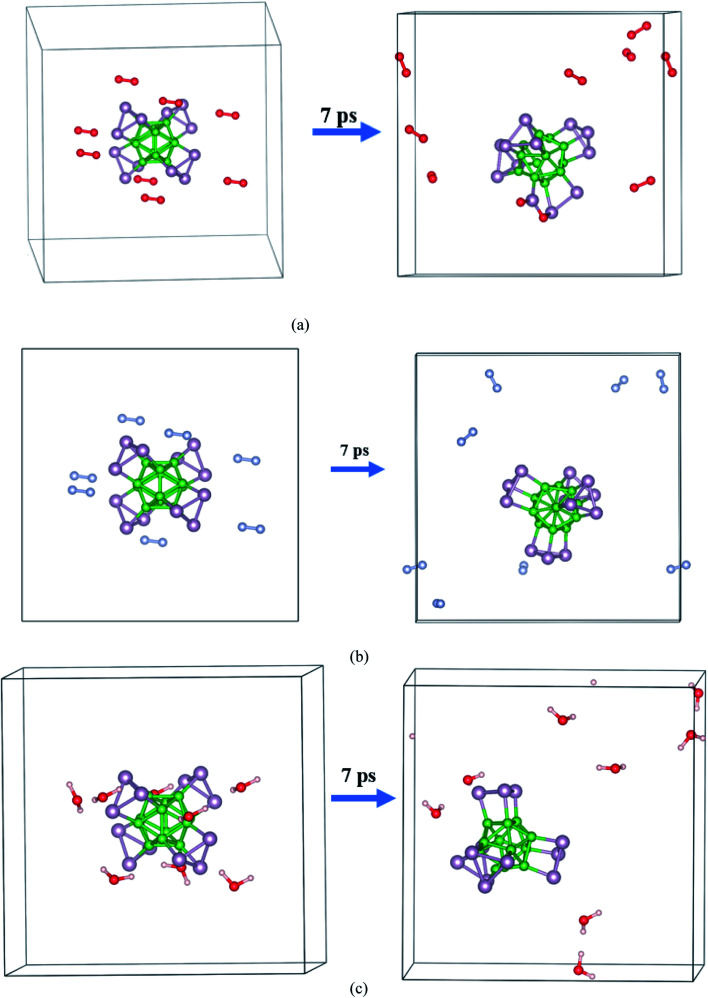
Initial and final snapshots of the α-B_12_P_12_ structure with gaseous phase (a) O_2_, (b) N_2_, and (c) H_2_O after 7 ps AIMD simulations at the temperature of 300 K.

**Fig. 6:**
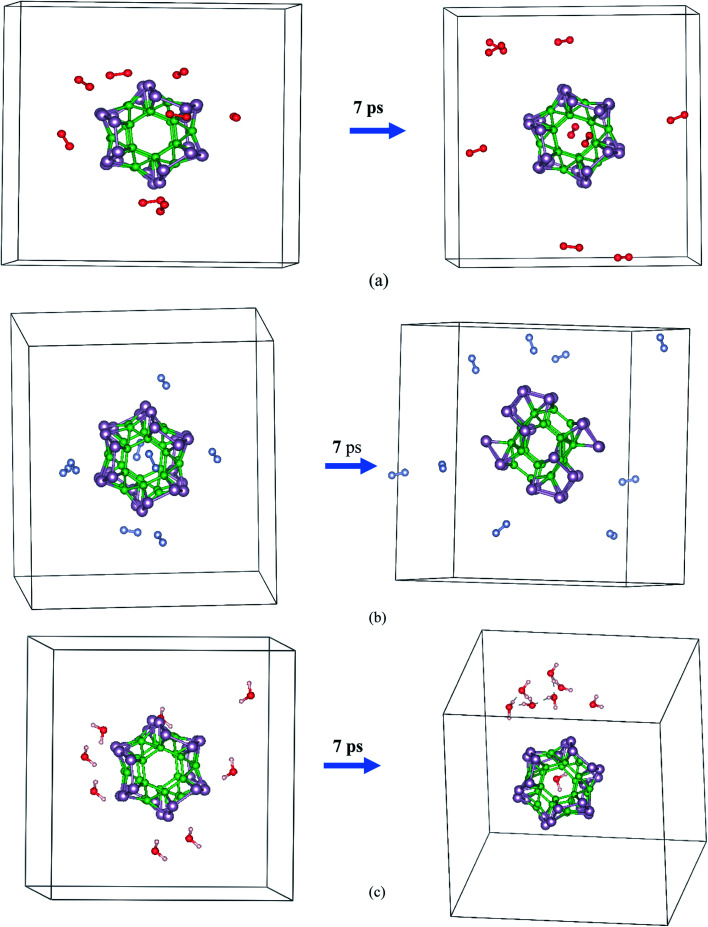
Initial and final snapshots of the α-B_24_P_24_ structure with gaseous phase (a) O_2_, (b) N_2_, and (c) H_2_O after 7 ps AIMD simulations at the temperature of 300 K.

The AIMD simulation was used to investigate the interaction between α-B_*n*_P_*n*_ and gaseous phase O_2_, N_2_, and H_2_O molecules at room temperature. As an initial model, randomly, eight O_2_, N_2_, and H_2_O molecules were added around the α-B_12_P_12_ andα-B_24_P_24_ clusters with 1 × 1 × 1 unit cells at an initial distance of 3.5–4 Å from the cluster surface (see Fig. 5 and 6). The lattice parameters of the optimized α-B_*n*_P_*n*_ structures at the PBE + D3 level are *a* = 19.4 Å, *b* = 19.4 Å, and *c* = 19.4 Å. The AIMD simulations were performed for 7 ps with a time step of 1.0 fs under 300 K. Based on our AIMD simulations, it can be seen that generally the α-B_*n*_P_*n*_ clusters have relatively high stability in the presence of O_2_, N_2_, and H_2_O molecules. Obviously, after 7 ps of contact, some O_2_ molecules dissociated into O atoms and chemisorbed onto the α-B_12_P_12_ surface. The P–O bonds with a bond length of about 1.524 Å are short and could be polar due to the difference in the electronegativity between the P and O atoms. Apart from the negative outcomes due to the α-B_12_P_12_ interaction with the O_2_ molecule, controlled passivation might lead to new stable structures for gas sensing. It should be reminded that the basic structure of the α-B_12_P_12_ cluster is also well maintained after 7 ps of contact. In contrast, the phenomena of O_2_ dissociation has not been found on the α-B_24_P_24_ surface in the AIMD simulation (see Fig. 6), which indicated the high oxidation resistance of the α-B_24_P_24_ structure. This result is very exciting and is different from the result we expected. Furthermore, the simulations identify that no interaction is observed between the α-B_12_P_12_/α-B_24_P_24_ structures with N_2_ and H_2_O molecules, which indicates the high stability of this type of structure. To confirm the chemical stability of the α-B_*n*_P_*n*_ clusters, the adsorption of O_2_, N_2_, and H_2_O gas molecules onto the α-B_*n*_P_*n*_ surfaces was performed. The adsorption energy (*E*_ads_; eV per gas molecule) can be calculated as follows.*E*_ads_ = (*E*_cluster–gas_ − (*E*_cluster_ + *nE*_gas_)/*n*where *n* is the number of gas molecules per unit cell. The *E*_cluster–gas_ term stands for the total energy of the α-B_*n*_P_*n*_ clusters after gas adsorption. The *E*_cluster_ and *E*_gas_ terms are the total energies of the isolated α-B_*n*_P_*n*_ cluster and the gas molecule, respectively. According to the above expression, the negative *E*_ads_ exhibits that complex formation is thermodynamically favorable. The calculated adsorption energy of the studied complexes is plotted in Fig. S5.† As seen from this figure, the adsorption energy of the O_2_ molecules onto the α-B_12_P_12_ surface is favorable (−42.93 kJ mol^−1^ per O_2_ molecule). According to the adsorption energy values, it is found that O_2_ physisorbs over α-B_12_P_12_ with an adsorption energy of about −43 kJ mol^−1^. These results are in good accordance with the results of the AIMD simulation, which were used to investigate the chemical stability α-B_*n*_P_*n*_ and O_2_, N_2_, and H_2_O gas molecules. Hence, it can be concluded that the structural integrity of α-B_*n*_P_*n*_ can be retained perfectly in the environment of gaseous phase O_2_, N_2_, and H_2_O at room temperature.

### 3.3. Electronic and optical properties

**Fig. 7:**
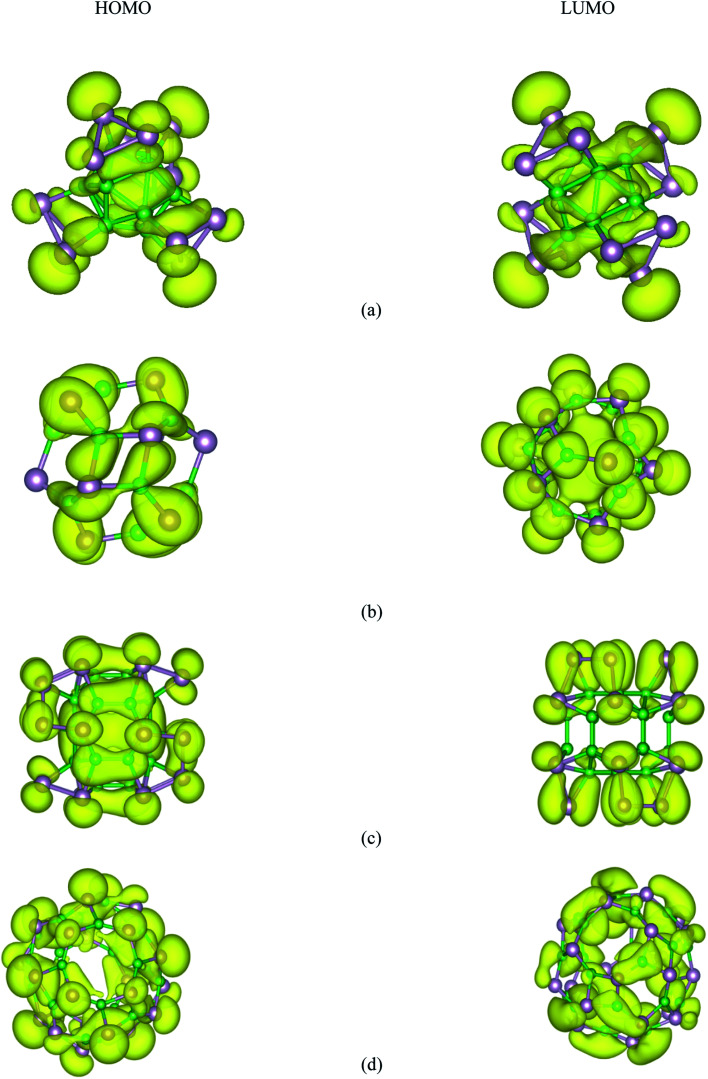
HSE06-DFT calculated contour plots of the HOMO and LUMOs orbitals of (a) α-B_12_P_12_, (b) β-B_12_P_12_, (c) α-B_24_P_24_, and (d) β-B_24_P_24_.

Based on the relaxed geometries, the electronic properties of the most stable structure, along with the first low-lying isomer of the B_*n*_P_*n*_ structures, were also investigated. The HOMO–LUMO (H–L) energy gap (*E*_gap_) is a fruitful parameter for examining the kinetic stability in molecules. The *E*_gap_ is the energy needed to excite the electron from the HOMO to the LUMO, where a large energy gap implies more kinetic stability and less reactivity. The HSE06 energy gaps of the ground-state structures and the first low-lying isomer of B_*n*_P_*n*_ are summarized in Table 2. As represented in Table 2, the H–L energy gaps of the α-B_*n*_P_*n*_ and β-B_*n*_P_*n*_ clusters are in the range of ∼0.67–3.2 eV, while the energy gaps in the β-B_*n*_P_*n*_ fullerene-like structures are higher than those of the corresponding α-B_*n*_P_*n*_ structures. It is revealed that α-B_12_P_12_ and α-B_24_P_24_ are semiconductor structures with the HSE06 band gap of 0.67 and 0.62 eV respectively, which is comparable with the band gap of the 2D-BP structure.^28^ In contrast, β-B_12_P_12_ and β-B_24_P_24_ fullerene-like structures have large band gaps of 3.2 and 2.98 eV, respectively. The small band gap value suggests modest chemical stability while the sizeable HOMO–LUMO energy gap is representative of the sufficient chemical stability of the studied clusters. The charge density distribution of the HOMO and LUMO for both the α-B_*n*_P_*n*_ and β-B_*n*_P_*n*_ structures are presented in Fig. 7. As seen, the HOMO and LUMO orbitals are generally composed of p-orbitals of phosphorus and boron atoms, and they have been distributed over the boron units and B–P bonds. In case of α-B_*n*_P_*n*_, each P atom with sp^3^ configuration has one lone pair of electrons in the fourth orbital (bonded with three neighbors of two P and one B atoms).^81^

Phosphorus has a higher electronegativity than the boron atom; hence, it is expected that the charge will be transferred from the B to the P atoms in the BP clusters. Bader charge analysis method is evaluated using the HSE06 functional^82^ and the corresponding values are collected in Table 2. As expected, boron atoms become slightly positively charged with the loss of electron. The amount of total charge transfer from the B atoms to the P atoms in the β-B_*n*_P_*n*_ structures is more significant than the corresponding α-B_*n*_P_*n*_ structures. Due to the charge transfer between B and P atoms, it is demonstrated that the B_*n*_P_*n*_ clusters have polar properties, especially for the β-B_*n*_P_*n*_ structures. It can be concluded that charge transfer between the B and P atoms demonstrates that electrostatic interactions have a significant effect on the stability of these types of structures.

**Fig. 8:**
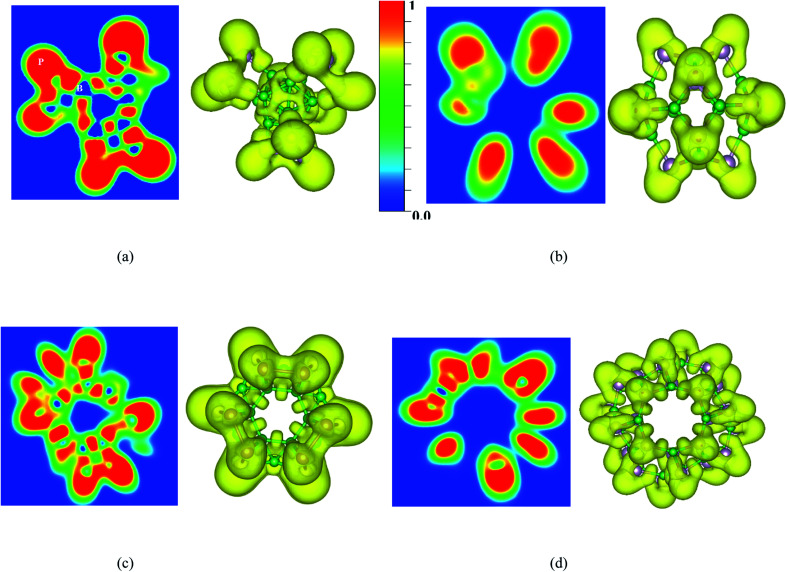
Contour and 3D plots of the electron localization function (ELF) (isovalue = 0.8 au) for (a) α-B_12_P_12_, (b) β-B_12_P_12_, (c) α-B_24_P_24_, and (d) β-B_24_P_24_ calculated by the HSE06 functional.

To understand more about the nature of bonds in the B_*n*_P_*n*_ structures, the electron localization function (ELF) calculated by VASP is also investigated. ELF can represent electron localization in a molecule and in the solid-state, and its values span from 0 to 1, which illustrates the degree of electron localization. Generally, the considerable value (∼1) of ELF is consistent with the strong covalent bonds or lone pair of electrons. On the other hand, a small value of ELF points out ionic or metallic bonds, and it shows the low electron density localization. Fig. 8 and S5† represent the ELF (isovalue = 0.8 au) of the B_*n*_P_*n*_ structures. From these results, it can be realized that electron localization around the P atoms is slightly more than that of the B atoms, which in good agreement with Bader charge analysis. Moreover, the line plots of the ELF values for the B–P bonds indicates the covalent nature of these bonds in the B_*n*_P_*n*_ structures (Fig. S6†).

**Fig. 9:**
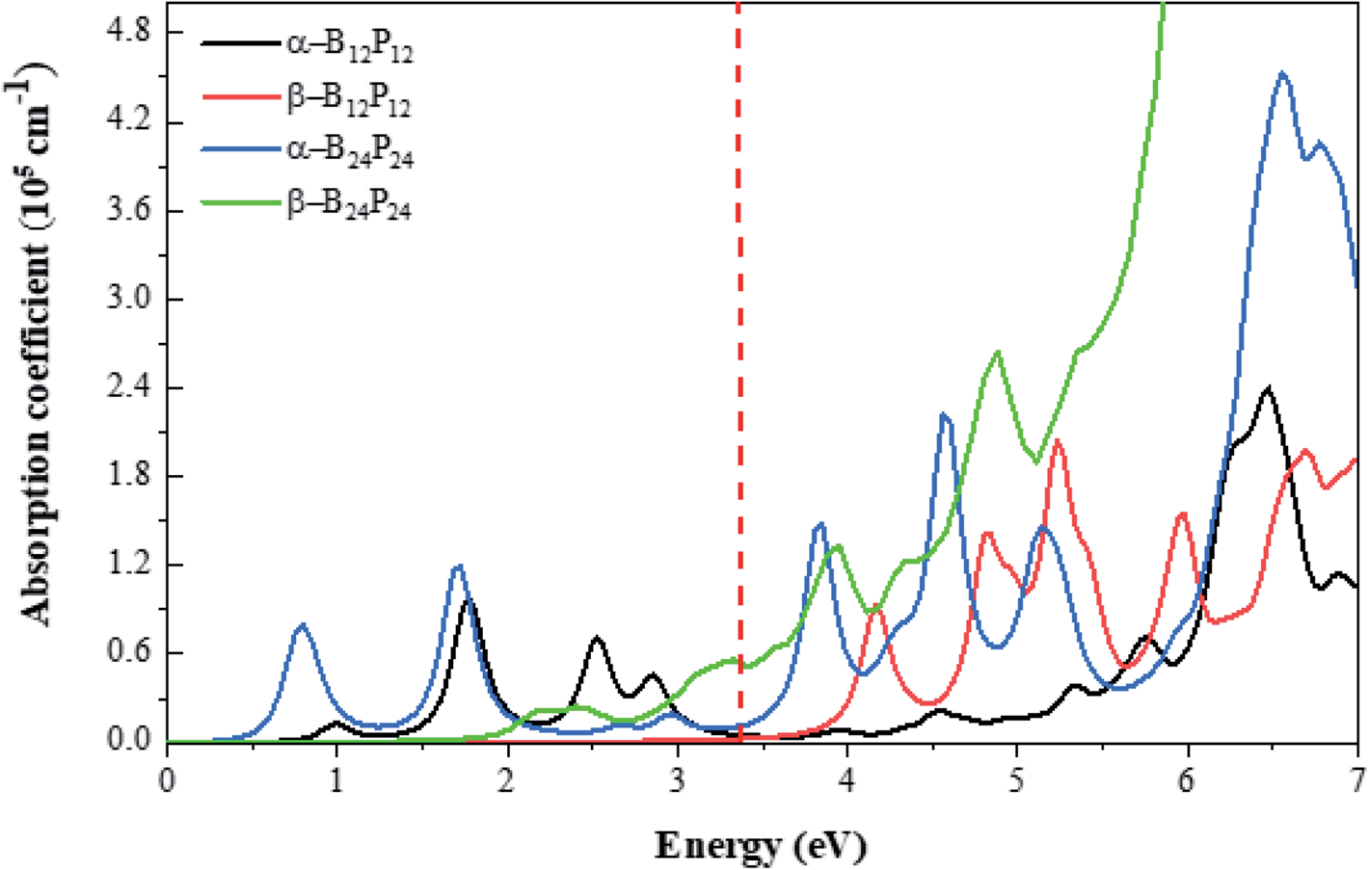
Optical absorption spectrum of (BP)_*n*_ with *n* = 12 and 24 clusters calculated by the HSE06 functional. The energy range corresponding to visible light is indicated by vertical red-dashed lines.

The high stability and moderate band gap feature of the investigated B_*n*_P_*n*_ clusters motivate us to explore the optical properties of the entitled structures. The optical properties of the B_*n*_P_*n*_ clusters have also been studied using the HSE06 functional. To investigate the performance under light, the optical absorption coefficient *α* was calculated according to the equation below.^75,83^2
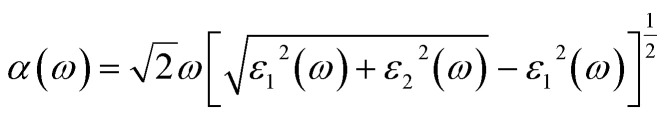
where *ε*_1_(*ω*) and *ε*_2_(*ω*) refer to the real and imaginary parts of the complex dielectric functions, respectively. As displayed in Fig. 9, B_*n*_P_*n*_ materials exhibit notable absorption in both visible and ultraviolet range with the absorption coefficient larger than 10^5^ cm^−1^, compared to many 2D-materials used as electronic and optoelectronic materials.^84^ According to Fig. 9, the optical absorption spectrum of both the structures illustrates a higher degree of absorption in the UV and visible region, which suggests the ability of the α-B_*n*_P_*n*_ and β-B_*n*_P_*n*_ structures to capture ultraviolet and visible light harvesting. In comparison, α-B_*n*_P_*n*_ exhibits stronger optical absorption than those corresponding to the β-B_*n*_P_*n*_ structures in the visible region.

**Fig. 10:**
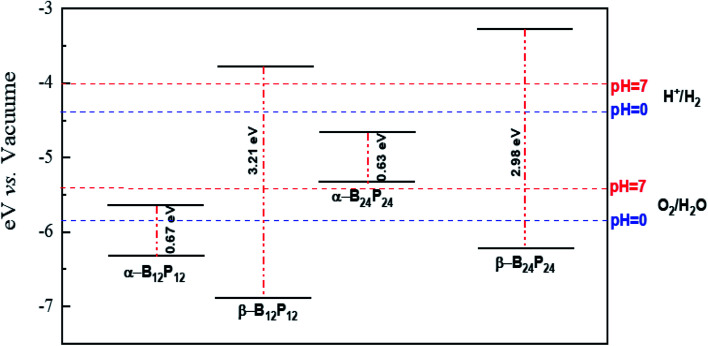
The local location of VBM and CBM with respect to the vacuum level, calculated with the HSE06 functional. The redox potentials of water splitting at pH = 0 (blue dashed line) and pH = 7 (red dashed line) are shown for comparison.

Besides, we focused on the potential applications of these compounds for photocatalytic water-splitting. First, the HSE06 position of the valence band maximum (VBM) and the conduction band minimum (CBM) was calculated from the difference between the electrostatic potential at the vacuum region and is shown in Fig. 10. As discussed above, the β-B_12_P_12_ and β-B_24_P_24_ structures have a band gap of 3.2 and 2.98 eV, respectively, which is located in the visible-light region. The optical absorption of the β-B_12_P_12_ and β-B_24_P_24_ fullerene-like structures, as shown in Fig. 9, represents that the β-B_12_P_12_ and β-B_24_P_24_ clusters have expectantly optical absorption in the visible-light region. The optical absorption spectrum of β-B_24_P_24_ starts from ∼550 nm, belonging to 2.25 eV, where the band gap of β-B_24_P_24_ is 2.98 eV. On the other hand, the band edge alignments concerning the oxygen and hydrogen evolution potential levels should be located at proper potentials. Our calculations demonstrate that the VBMs and CBMs of the β-B_12_P_12_ and β-B_24_P_24_ clusters meet both conditions for photocatalytic water splitting. As seen from Fig. 10, their valence bands lie at more positive potentials than the water oxidation potential (O_2_/H_2_O potential) and their conduction bands (CBMs) are more negative than the hydrogen reduction potential (H^+^/H_2_ potential). These results clearly show that the β-B_*n*_P_*n*_ structures have great potential for applications as a visible-light photocatalyst for water splitting. In contrast, the strong absorption coefficient of the α-B_24_P_24_ (which can reach a higher order of 5 × 10^5^ cm^−1^) and α-B_12_P_12_ systems is comparable to that of the organic perovskite solar cell. These results reveal that α-B_24_P_24_ and α-B_12_P_12_ have great potential for applications as optical absorbent materials in solar cells and optoelectronic devices.

### 3.4. NLO properties

Based on the research conducted to investigate the ability of (XY)_*n*_ fullerene-like clusters as NLO materials,^31,38,85,86^ it has motivated us to investigate the second-order NLO properties of both α-B_*n*_P_*n*_ and β-B_*n*_P_*n*_ structures. Second-order NLO materials that can alter the wavelength of lasers are of considerable interest because of the potential of utilization of such materials in optical switching, optical computing, and photonic technology.^31,38,85,86^ Quartz crystal (α-SiO_2_) is a first second-order NLO material, as demonstrated by Franken *et al.* in 1961.^87,88^ In most recent studies, the ability of metal encapsulated (XY)_*n*_ nano-cages (X = B, Al, and Y = N, P) as NLO materials has been considered. They found that the (XY)_*n*_ nano-cages have zero dipole moment and zero hyperpolarizability. It should be noted that none of these studies have examined the second-order NLO properties. Herein, for both α-B_*n*_P_*n*_ and β-B_*n*_P_*n*_ structures, the average polarizability (*α*), first hyper-polarizabilities (*β*_0_), and second hyperpolarizability (*γ*) were calculated at the CAM-B3LYP/def2-tzvp level of theory using the Gaussian 09 package.^89^ As seen in Table 3, the mean polarizabilities (*α*) of α-B_*n*_P_*n*_ are higher than those of the corresponding β-B_*n*_P_*n*_ structures. The maximum value of α is related to the α-B_24_P_24_ structure with 965.24 a.u., which is higher than that of the many encapsulated metal–(XY)_*n*_ nano-cages.^31,86^ Furthermore, our calculations proved that both α-B_*n*_P_*n*_ and β-B_*n*_P_*n*_ structures have nearly zero first hyper-polarizabilities (*β*_0_). The second hyper-polarizabilities (*γ*) of α-B_*n*_P_*n*_ and β-B_*n*_P_*n*_ are also summarized in Table 3. The second hyper-polarizabilities of all the predicted structures are higher than the experimental values of *γ* for C_60_ and C_70_ fullerenes,^90,91^ and comparable to that of the best NLO materials reported in the literature. On the other hand, α-B_12_P_12_ and α-B_24_P_24_ have larger *γ* values than the corresponding β-B_*n*_P_*n*_ clusters. The *γ* value of α-B_24_P_24_ is 29 times greater than the second hyper-polarizability value of the β-B_24_P_24_ clusters. In conclusion, the strong interactions, effective intermolecular charge transfer, and cluster shape affect the magnitude of the second hyper-polarizabilities of the α-B_12_P_12_ and α-B_24_P_24_ structures. Furthermore, the α-B_12_P_12_ and α-B_24_P_24_ clusters show not only high stability but also have large second hyperpolarizability. Hence, they are anticipated to be excellent potential unprecedented candidates for second-order NLO materials.

**Table 3:** CAM-B3LYP/def2-tzvp results for the average polarizability (*α* in a.u.), first hyperpolarizability (*β*0 in a.u.), and the average values of the second hyperpolarizability (*γ* in a.u.) of the (BP)_*n*_ clusters

Type	〈*α*〉	*β*_0_	〈*γ*〉
α-B_12_P_12_	476	10.11	79 172.77
β-B_12_P_12_	393.22	0.05	31 780.03
α-B_24_P_24_	965.24	385.97	2 581 589
β-B_24_P_24_	867.12	0.78	89 540.24

## 4. Conclusion

In this work, we explored the new stable structure of B_*n*_P_*n*_ (*n* = 12, 24) clusters with a modern method of crystal structure prediction, USPEX conjugated with spin-polarized first-principles calculations. Our prediction demonstrates that the most stable structures of B_12_P_12_ and B_24_P_24_ are α-B_12_P_12_ and α-B_24_P_24_. Based on the relaxed geometries of the α-B_*n*_P_*n*_ structures, each phosphorus atom is doped into a boron atom while the B atoms form a B_*n*_ unit. In contrast, the first low-lying β-B_*n*_P_*n*_ isomers of the (BP)_*n*_ clusters are shaped like fullerenes, where each boron atom is bonded to each phosphorus atom to make cage structures. The calculated binding energies for these novel structures are in the range from −4.723 to −4.803 eV per atom, which is much better or comparable to that of other 2D materials and clusters, indicating their excellent stability and possible experimental synthesis. Furthermore, α-B_*n*_P_*n*_ structures are more stable than the corresponding β-B_*n*_P_*n*_ structures, and the maximum binding energy of −4.803 eV per atom belongs to the α-B_24_P_24_ structure. Based on our AIMD simulations, it was found that α-B_*n*_P_*n*_ structures have relatively high stability in the presence of O_2_, N_2_, and H_2_O molecules, in particular, α-B_24_P_24_ shows superior oxidation resistance. In the case of thermal stability, α-B_*n*_P_*n*_ and β-B_*n*_P_*n*_ exhibit better thermal stability, where the upper limit temperature that the structures can tolerate is 900 K.

It is revealed that α-B_12_P_12_ and α-B_24_P_24_ are semiconductor structures with the HSE06 band gap of 0.67 and 0.62 eV, respectively, which is comparable with the band gap of the 2D-BP structure. In contrast, β-B_12_P_12_ and β-B_24_P_24_ fullerene-like structures have large band gaps of 3.2 and 2.98 eV, respectively. The calculated Bader charges illustrate their ionic characters with charge transfers from the B to P atoms. The electronic properties of these novel compounds illustrate a higher degree of absorption in the UV and visible-region with an absorption coefficient larger than 10^5^ cm^−1^, which suggests a wide range of opportunities for advanced optoelectronic applications. The β-B_*n*_P_*n*_ phase has suitable band alignments in the visible-light excitation region, which will produce enhanced photocatalytic water splitting. On the other hand, α-B_*n*_P_*n*_ structures with high absorption coefficient exhibit large second hyperpolarizability, which are expected to have excellent potential as second-order NLO materials.

## Conflicts of interest

There are no conflicts to declare.

## Supplementary Material

NA-003-D0NA01040E-s001
