# Supplementary material for: Prediction of unexpected BnPn structures: promising materials for non-linear optical devices and photocatalytic activities
Source: Nanoscale Adv. 2021 Mar 26;3(10):2846–61. doi: 10.1039/d0na01040e (PMC9417267; doi:10.1039/d0na01040e)
Supplement: NA-003-D0NA01040E-s001 [file NA-003-D0NA01040E-s001.pdf]

Electronic Supplementary Material (ESI) for Nanoscale Advances.
This journal is © The Royal Society of Chemistry 2021

Supplementary Information for:

Prediction of Unexpected B_nP_n Structures: A Promising Materials for Nonlinear Optical Devices and Photocatalytic Activities

Zabihollah Mahdavifar*

Department of Chemistry, Faculty of Science, Shahid Chamran University of Ahvaz, Ahvaz, Iran

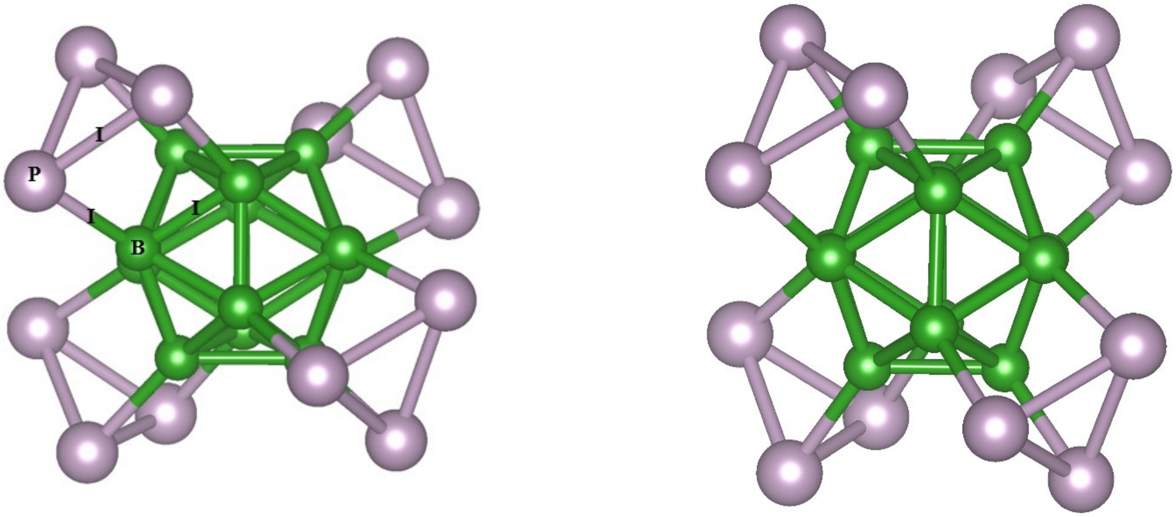

(a)

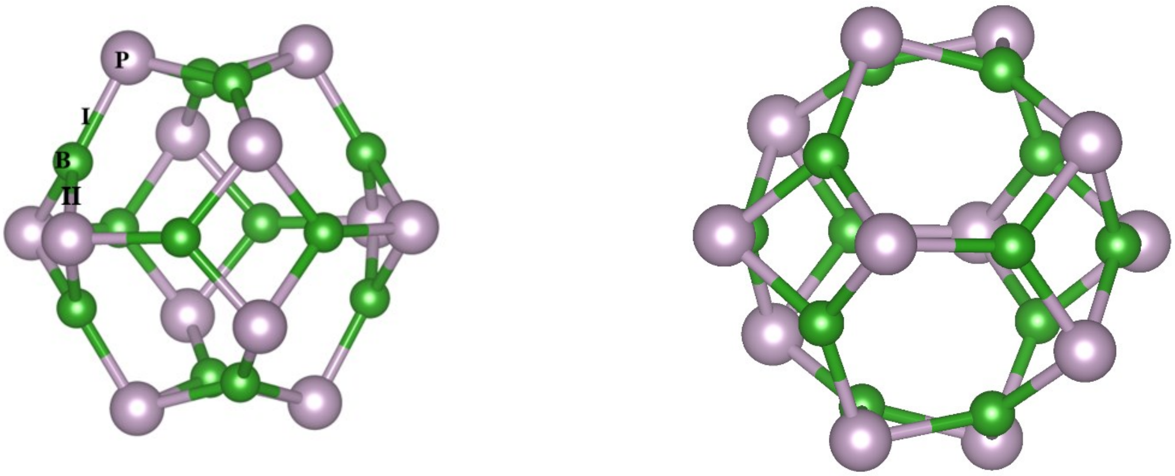

(b)

Fig. S1. Relaxed geometry of the most stable structure and first low-laying energy isomer of (a) α-$B_{12}P_{12}$ and β-$B_{12}P_{12}$ clusters obtained at HSE06 level of theory.

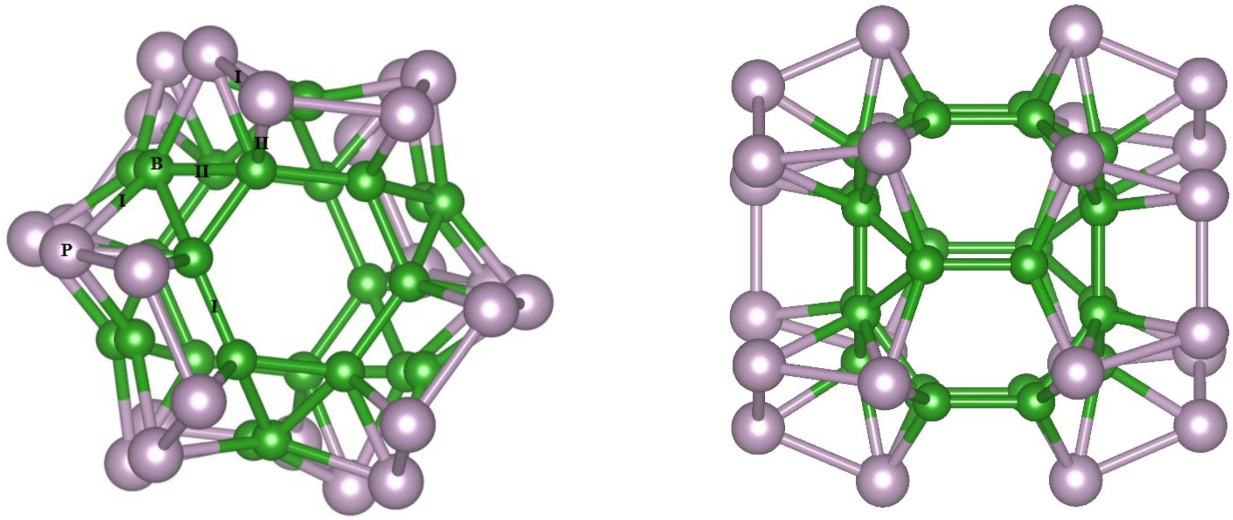

(a)

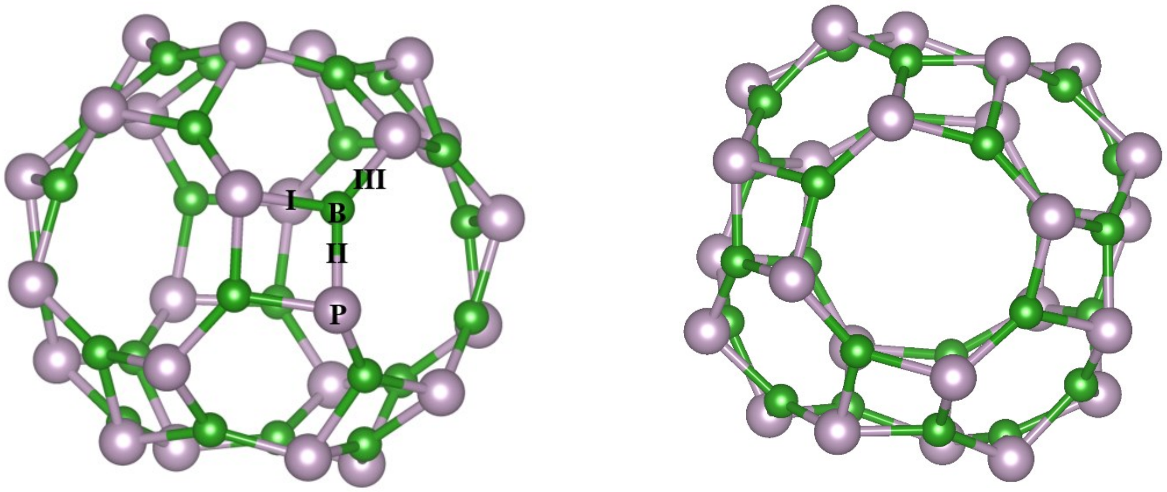

(b)

Fig. S2. Relaxed geometry of the most stable structure and first low-laying energy isomer of (a) α-$B_{24}P_{24}$ and β-$B_{24}P_{24}$ clusters obtained at HSE06 level of theory.

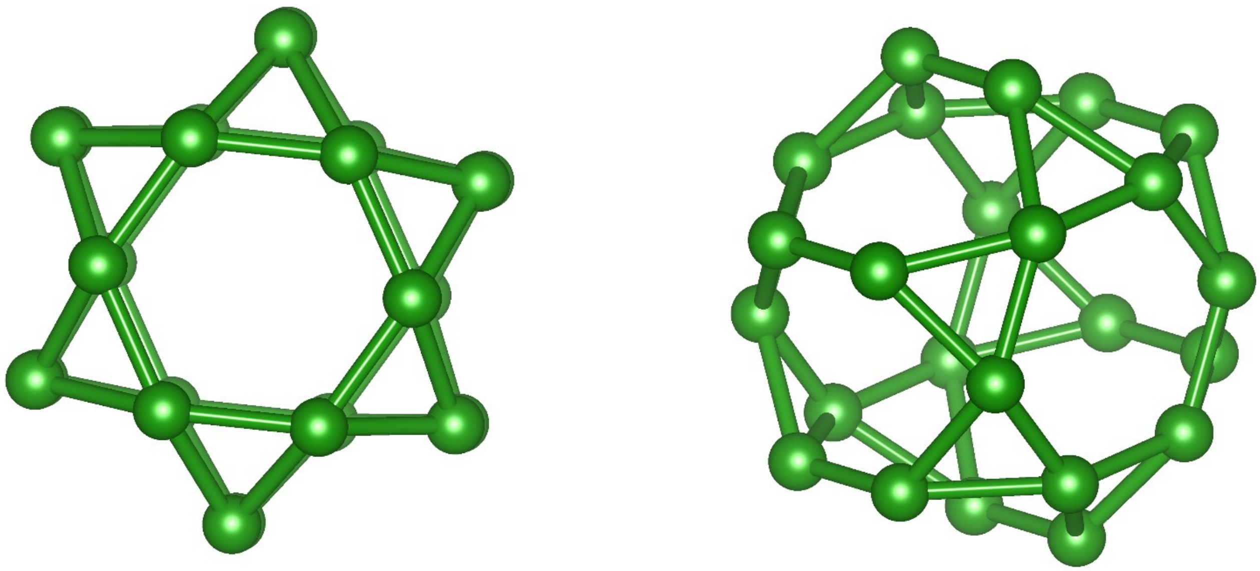

Fig. S3: Two different views of the B_{24} unit without P atoms of α-$B_{24}P_{24}$.

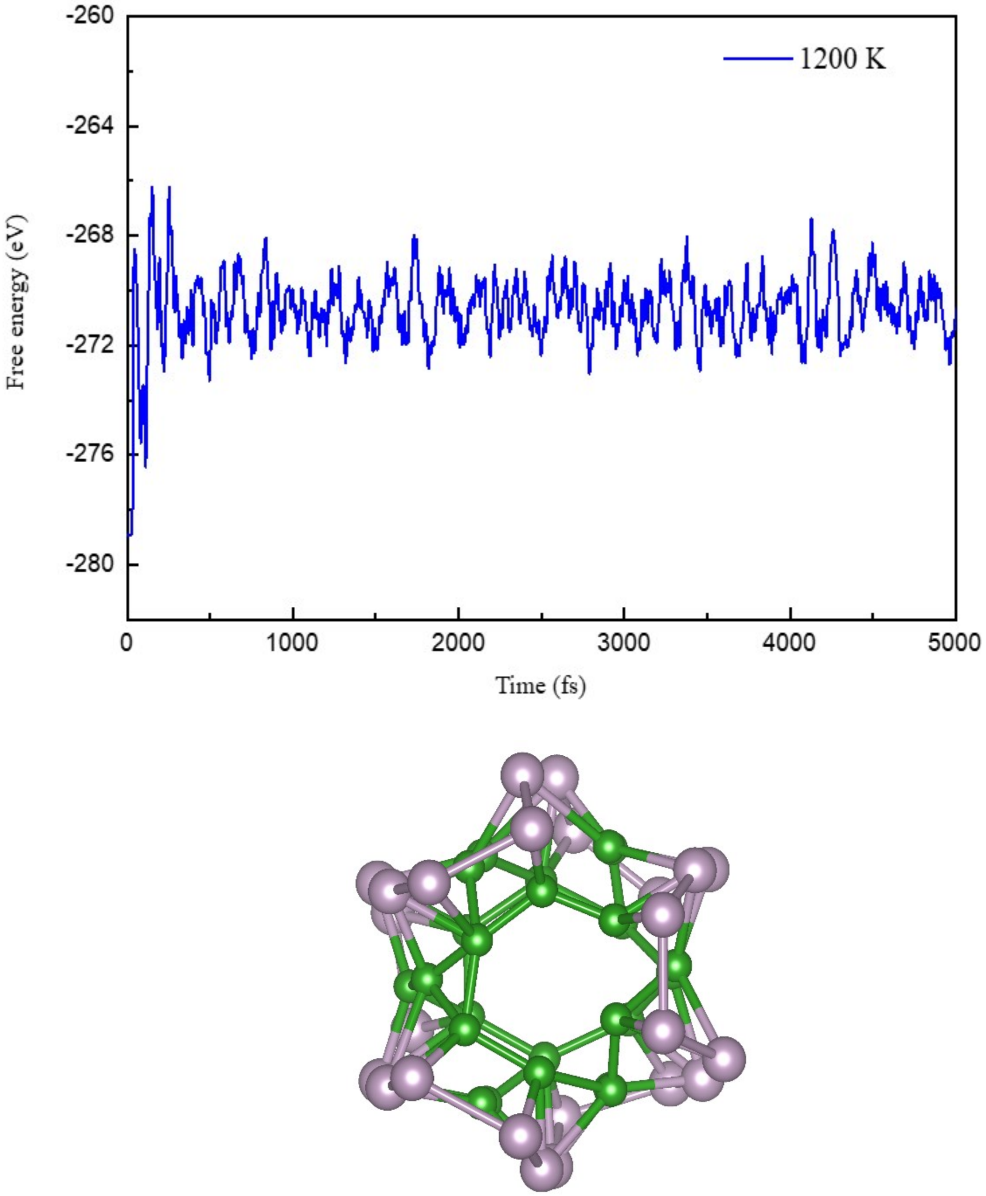

Fig. S4. The snapshot of the most stable structures for a time of 5 ps of α-$B_{24}P_{24}$. The temperature of the AIMD was set to 1200 K. the Bond breaking is observed at 6-memberd ring.

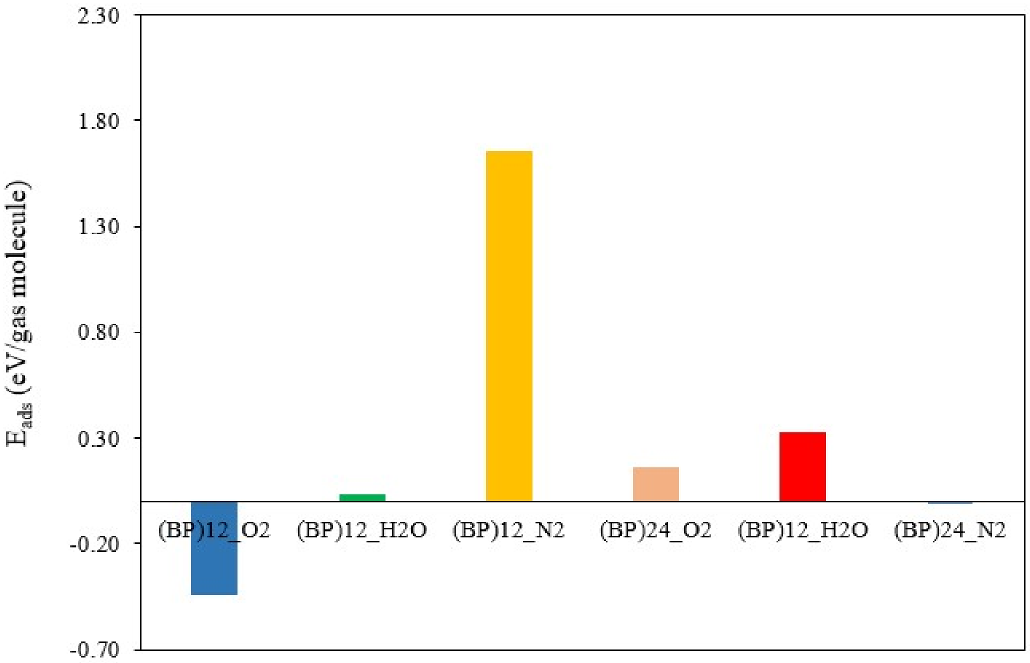

Fig. S5: Calculated adsorption energy (E_{ads}; eV/gas molecule) of eight O_2, N_2 and H_2O molecules adsorbed onto the α-$B_{12}P_{12}$ and α-$B_{24}P_{24}$ structures.

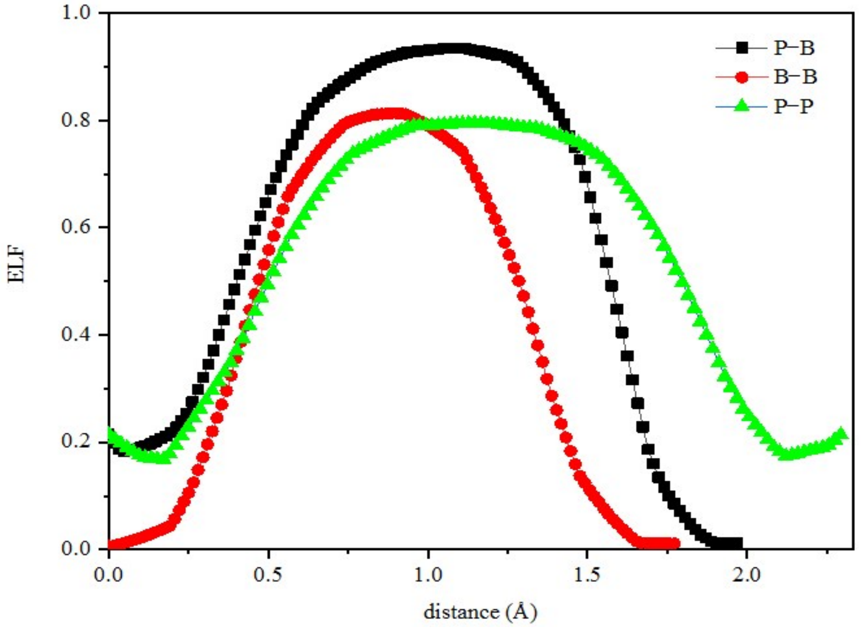
1.0
0.8
0.6
0.4
0.2
0.0
ELF
0.0
0.5
1.0
1.5
2.0
distance (Å)
P–B
B–B
P–P

(a)

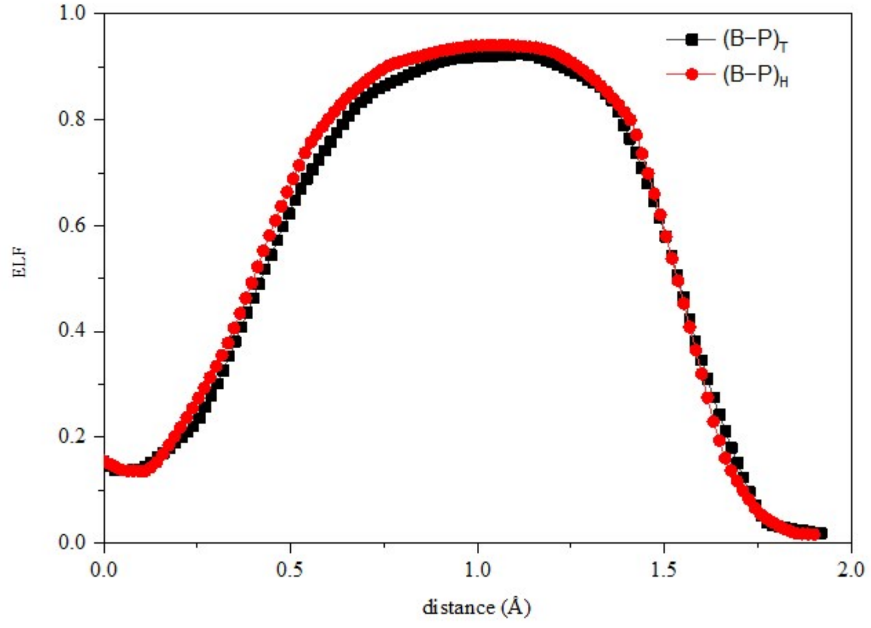
1.0
0.8
0.6
0.4
0.2
0.0
ELF
0.0
0.5
1.0
1.5
2.0
distance (Å)
(B–P)T
(B–P)H

(b)

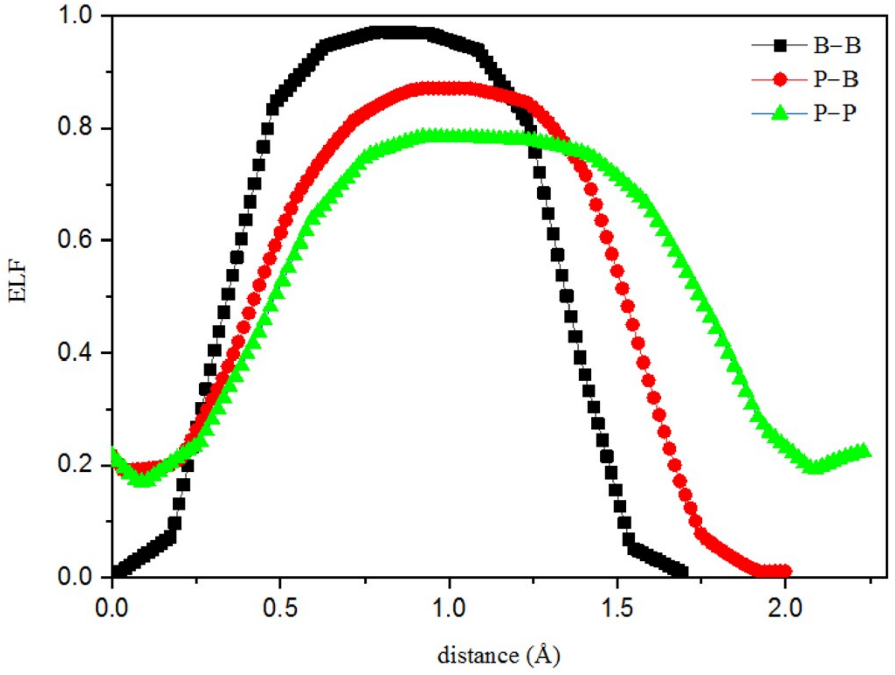

(c)

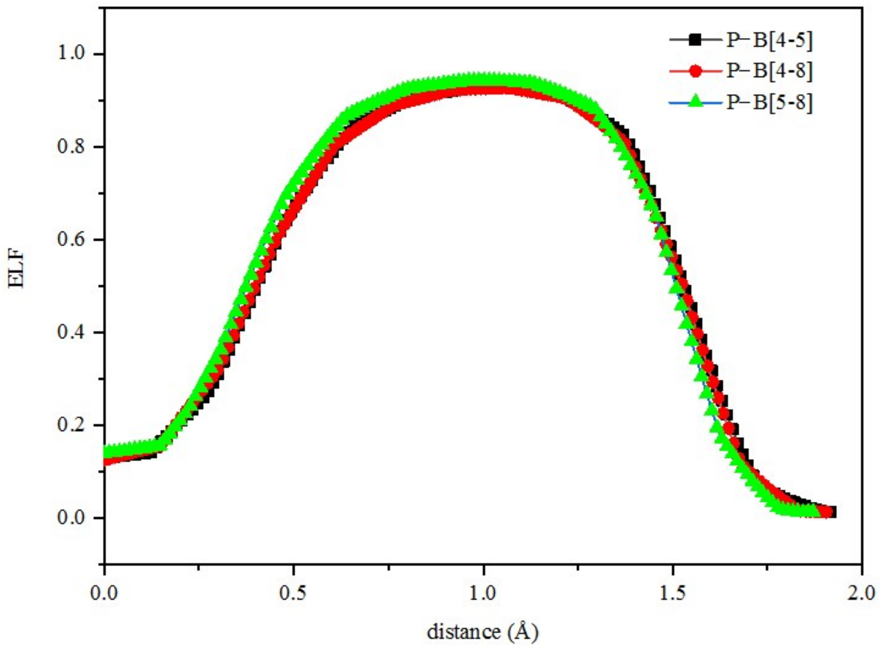

(d)

Fig. S5: Line plots of ELF for (a) α-$B_{12}P_{12}$, (b) β-$B_{12}P_{12}$, (c) α-$B_{24}P_{24}$ and (d) β-$B_{24}P_{24}$ clusters computed at HSE06 level of theory.
